# Multi-country Survey Revealed Prevalent and Novel F1534S Mutation in Voltage-Gated Sodium Channel (VGSC) Gene in *Aedes albopictus*

**DOI:** 10.1371/journal.pntd.0004696

**Published:** 2016-05-04

**Authors:** Jiabao Xu, Mariangela Bonizzoni, Daibin Zhong, Guofa Zhou, Songwu Cai, Yiji Li, Xiaoming Wang, Eugenia Lo, Rebecca Lee, Roger Sheen, Jinhua Duan, Guiyun Yan, Xiao-Guang Chen

**Affiliations:** 1 Department of Parasitology, School of Public Health and Tropical Medicine, Southern Medical University, Guangzhou, China; 2 Program in Public Health, University of California, Irvine, Irvine, California, United States of America; 3 Department of Biology and Biotechnology, University of Pavia, Pavia, Italy; 4 Department of Vector Control, Centers for Disease Control and Prevention of Guangdong Province, Guangzhou, China; Mahidol University, THAILAND

## Abstract

**Background:**

*Aedes albopictus* is an important dengue vector because of its aggressive biting behavior and rapid spread out of its native home range in Southeast Asia. Pyrethroids are widely used for adult mosquito control, and resistance to pyrethroids should be carefully monitored because vector control is the only effective method currently available to prevent dengue transmission. The voltage-gated sodium channel gene is the target site of pyrethroids, and mutations in this gene cause knockdown resistance (*kdr*). Previous studies reported various mutations in the voltage-gated sodium channel (VGSC) gene, but the spatial distribution of *kdr* mutations in *Ae*. *albopictus* has not been systematically examined, and the association between *kdr* mutation and phenotypic resistance has not been established.

**Methods:**

A total of 597 *Ae*. *albopictus* individuals from 12 populations across Asia, Africa, America and Europe were examined for mutations in the voltage-gated sodium channel gene. Three domains for a total of 1,107 bp were sequenced for every individual. Two populations from southern China were examined for pyrethroid resistance using the World Health Organization standard tube bioassay, and the association between *kdr* mutations and phenotypic resistance was tested.

**Results:**

A total of 29 synonymous mutations were found across domain II, III and IV of the VGSC gene. Non-synonymous mutations in two codons of the VGSC gene were detected in 5 populations from 4 countries. A novel mutation at 1532 codon (I1532T) was found in Rome, Italy with a frequency of 19.7%. The second novel mutation at codon 1534 (F1534S) was detected in southern China and Florida, USA with a frequency ranging from 9.5–22.6%. The WHO insecticide susceptibility bioassay found 90.1% and 96.1% mortality in the two populations from southern China, suggesting resistance and probable resistance. Positive association between *kdr* mutations with deltamethrin resistance was established in these two populations.

**Conclusions:**

Two novel *kdr* mutations, I1532T and F1534S were found in *Ae*. *albopictus*. This is the first report of I1532T mutations in Italy and F1534S mutation in China and US. Significant association between *kdr* mutation and protection from deltamethrin raised the possibility that *kdr* mutation may be a viable biomarker for pyrethroid resistance surveillance in *Ae*. *albopictus*. The patchy distribution of *kdr* mutations in *Ae*. *albopictus* mosquitoes calls for developing global surveillance plan for pyrethroid resistance and developing countermeasures to mitigate the spread of resistance.

## Author Summary

*Aedes albopictus* is a major dengue and Chikungunya vector and highly invasive. In the absence of effective treatment for the arbovirus infections, vector control is the only viable option. Pyrethroids are the most widely used insecticide for vector control programs due to low mammalian toxicity and rapid knockdown action. Extensive and prolonged use of pyrethroids imposes selection pressure on mosquito populations and eventually increases the potential of resistance. Monitoring pyrethroid resistance is essential to effective management of resistance. The voltage-gated sodium channel gene is the target site of pyrethroids, and mutations in this gene result in knockdown resistance (*kdr*). Previous studies reported various mutations in the VGSC gene, but the spatial distribution of *kdr* mutations in *Aedes albopictus* has not been systematically examined, and the association between *kdr* mutation and resistance has not been established. In the present study, we examined *kdr* mutation distribution in 12 populations from Asia, Africa, America and Europe. We found two novel and abundant *kdr* mutations, and established significant positive association between *kdr* mutations with deltamethrin resistance. This finding raised the possibility that *kdr* mutation may be a viable biomarker for pyrethroid resistance surveillance in *Ae*. *albopictus*. The patchy distribution of *kdr* mutations in *Ae*. *albopictus* mosquitoes calls for developing global surveillance plan for pyrethroid resistance and developing countermeasures to mitigate the spread of resistance.

## Introduction

*Aedes albopictus*, also known as Asian tiger mosquito, is notorious for its ability to transmit a number of arboviruses including dengue, Chikungunya and Zika virus as well as filarial nematodes [1–11]. The aggressive dispersal and thus world-wide invasiveness in recent years [5,6], in addition to increased vector competence to Chikungunya viruses [9,10,12–15], have proved high public health impact of *Ae*. *albopictus*. For instance, global spread of *Ae*. *albopictus* is linked to Zika virus outbreaks in urban areas of central Africa, Asia and the Pacific [16–18].

At present, there are neither effective vaccines nor therapeutic treatments targeted for viruses vectored by *Ae*. *albopictus*, making vector population control the only option to limit disease transmission [1,2,4–6,16]. Current vector control strategies primarily rely on the source reduction of larval breeding sites and use of insecticides targeting the larval and adult stages [4,16–25]. Among the four major synthetic insecticides groups, pyrethroids are the most widely used adulticide due to their low mammalian toxicity and their rapid knockdown effect [22–24,26]. Pyrethroids have been intensively used for space spray to control *Aedes* mosquitoes and dengue transmission [1,10,26–30]. Extensive and prolonged use of pyrethroids imposes selection pressure on *Ae*. *albopictus* populations and eventually increases the potential of resistance [2,3,9,10,25–28,31].

Pyrethroids target the VGSC gene, also known as voltage-gated sodium channel (VGSC) of insect neurons [28,32,33]. Generally among insects, there are two major mechanisms for conferring resistance against these insecticides. One is increased metabolic detoxification of insecticides, which is the most common form of resistance mechanism because of the higher expression or presence of more efficient detoxification enzymes [34]. The other known mechanism is reduced target site sensitivity resulting from non-synonymous mutations in the VGSC gene, leading to single amino acid substitutions, which has been shown to be correlated to phenotypic resistance to pyrethroids [35]. This form of resistance, known as knockdown resistance (*kdr*), has been observed in a number of insects, including *Anopheles gambiae* [33,34], *An*. *sinensis* [35,36], *Culex quinquefasciatus* [37,38] and *Ae*. *aegypti* [32,39].

In *An*. *gambiae*, L1014F and L1014S in domain II of Subset 6 (IIS6) of the voltage-gated sodium channel (VGSC) gene are the most well-known mutations related to pyrethroids and DDT resistance [33,34,37,38]. In *Ae*.*aegypti*, V1016G and V1016I mutation in IIS6 are positively related to pyrethroid resistance, and F1534C was found conferring pyrethroid resistance [40–42]. In *Drosophila melanogaster*, M1524I substitution has been associated with knockdown resistance [43] and in other arthropods and mammals, F1538I mutation was associated with reduced sensitivity to deltamethrin [44,45]. In *Ae*. *aegypti*, V1016I and V1016G mutations alter VGSC configuration and subsequently prevent insecticide binding. Codon 1530 and 1529 on IIIS6 of VGSC have been proposed to be r sensitivity to pyrethroids [46,47]. Two residues nearby codons 1535 and 1538 have been implicated in resistance to pyrethroids in other insect species [48,49]. For *Ae*. *albopictus*, F1534C was first reported in Singapore in 2011 [28], and the same mutation was subsequently found in Malaysia as well as in the United States but with a different allele (F1534L) [25, 43]. Along with the emergence and spread of *kdr* mutations, recent studies have demonstrated pyrethroid resistance in *Ae*. *albopictus* adults from different parts of southeast Asia such as Malaysia and Thailand and from Central Africa [1,25,27,31,50,51]. Previous studies examined *kdr* mutations in limited number of populations, systematic examination of *kdr* mutations in *Ae*. *albopictus* populations from a broader geographic range would provide important information on *kdr* mutation distribution and potential risk of resistance spread.

In the present study, we examined mutations in the VGSC gene of *Ae*. *albopictus* across Asia, Europe, and North America, encompassing almost the entire range of its distribution. In addition, the association between *kdr* mutation and phenotypic resistance was assessed in a subset of populations to provide a deeper insight into the role of *kdr* mutations on pyrethroid resistance.

## Materials and Methods

### Mosquito sampling sites

*Aedes albopictus* samples were collected between 2011 and 2014 in 12 sites from 6 countries (S1 Table). These sites were selected based on the global distribution of *Ae*. *albopictus* and willingness of in-country collaborators. These sampling sites included the native home range in Southeast Asia (i.e. Guangzhou, Shenzhen [China], Nagasaki [Japan] and Serangoon [Singapore]), and derived populations (i.e. Hawai’i [USA], La Reunion [France], California, Texas, Florida [USA]], Arco, Rome [Italy], and Athens [Greece]). At all sampling sites, pyrethroids were the commonly used insecticide for vector control and agricultural pest control [52,53]. Historically, organophosphates were used for vector control since 1950s [5,54,55] in these sites. For each sample site, immature *Ae*. *albopictus* (larvae and pupae) were collected from more than 50 different aquatic habitats, such as discarded plastic containers, flower pots and used tires except that in La Reunion adult mosquitoes were collected using the BG-sentinel trap (Biogents, Regensburg, Germany). In each site mosquitoes were collected in one time point as indicated in Table 1. The field collected larvae/pupae were reared to adults and preserved for subsequent DNA analysis.

**Table 1:** Genotyping results of F1534 alleles in the voltage-gated sodium channel gene in 12 *Aedes albopictus* populations.

Population	N	F1534 genotype							Allele frequencies (%)				P* (HWE)
		FF	FC	CC	FS	SS	FL	LL	F1534	F1534C	F1534S	F1534L	
Nagasaki, Japan	44	44	0	0	0	0	0	0	100	0	0	0	-
Guangzhou, China	62	37	0	0	2	5	4	14	63.5	0	9.5	25.4	<0.0001
Shenzhen, China	51	30	0	0	11	6	3	1	72.6	0	22.6	4.9	<0.0001
Singapore, Singapore	40	40	0	0	0	0	0	0	100	0	0	0	-
La Reunion, France	56	56	0	0	0	0	0	0	100	0	0	0	-
Arco, Italy	48	47	0	0	0	0	1	0	99.0	0	0	1.0	-
Rome, Italy	76	76	0	0	0	0	0	0	100	0	0	0	-
Athens, Greece	62	39	16	7	0	0	0	0	75.8	24.2	0	0	<0.05
California, USA	58	58	0	0	0	0	0	0	100	0	0	0	-
Texas, USA	32	32	0	0	0	0	0	0	100	0	0	0	-
Hawai’i, USA	26	26	0	0	0	0	0	0	100	0	0	0	-
Florida, USA	42	32	0	0	10	0	0	0	88.1	0	11.9	0	>0.05
Laboratory	60	60	0	0	0	0	0	0	100	0	0	0	-

HWE = Hardy-Weinberg equilibrium

* P refers to P value for chi-square test.

### DNA extraction and *kdr* mutations detection

Genomic DNA was extracted from individual mosquitoes using the SYBR Green Extract-N-Amp Tissue PCR Kit (Sigma Aldrich) following the manufacturer’s protocol. Extracted DNA was stored at 4°C or used immediately for PCR.

All the specimens were identified as *Ae*. *albopictus* using PCR with species-specific primers for the ribosomal internal transcribed spacer (ITS1 and ITS2) and 18S rDNA regions [45]. A total of 597 *Ae*. *albopictus* mosquitoes, ranging from 26–76 individuals per population, were subjected to *kdr* genotyping. Portions of domains II, III, IV of the VGSC gene were amplified, following protocols and primers developed by Kasai *et al* [28] (covering 989, 1011, 1016 and 1534 codon positions). PCR products were purified with ExoSAP-IT (USB, Cleveland, Ohio, USA) according to the manufacturer’s protocol and directly sequenced by Genewiz Inc. (South Plainfield, NJ). The sequences were aligned and analyzed using BioEdit (http://www.mbio.ncsu.edu/BioEdit/bioedit.html) and Codon code (http://www.codoncode.com/).

### Insecticide susceptibility bioassay

To determine resistance level of *Ae*. *albopictus* in the field, we conducted pyrethroid susceptibility bioassay in two populations from southern China (Shenzhen and Guangzhou). Briefly, in each location ~8,000 larvae were collected from 400 natural habitats and reared to adults in insectary. All specimens were identified to species by morphology. Adult females 3–5 days post emergence were subjected to insecticide susceptibility test against 0.05% deltamethrin following the standard WHO tube test protocol [10]. Control tests were performed using silicone oil, pre-impregnated papers. Adult bioassays were conducted with 20–25 mosquitoes per replicate, and 8–20 replicates per population. The number of mosquitoes being knocked down was recorded every 10 minutes during the 60 min exposure period. Mortality was scored after 24 hr recovery period. Overall, 420 female adults from Guangzhou and 150 individuals from Shenzhen were subjected to susceptibility bioassay. The susceptible Foshan strain of *Ae*. *albopictus* originated from Foshan city (40 Km away from Guangzhou) and has been maintained in the laboratory since 1981 with no insecticide exposure, was used as a susceptible reference population. Insecticide susceptibility bioassay was performed in China only, but not in other countries due to logistic constraints.

To establish association between *kdr* mutations and phenotypic resistance, we screened 2017 female adults from Guangzhou and 1350 from Shenzhen for deltamethrin resistance using the standard WHO tube bioassay. Here resistant individual is defined as a mosquito being alive after the 24 hr recovery period, and susceptible mosquito is defined as being dead after the 24 hr recovery period. This definition of resistance is reasonable because the 0.05% deltamethrin diagnostic dose kills 99.9% susceptible mosquitoes [56]. This screening yielded 79 and 115 resistant mosquitoes from Guangzhou and Shenzhen respectively. All these resistant mosquitoes and 153 susceptible mosquitoes were genotyped for *kdr* mutation at 1534 codon by direct sequencing.

### Statistical analysis

For *kdr* mutation survey in multiple populations, mutation frequencies at each codon were calculated for each population. Frequencies of synonymous and non-synonymous mutations were presented. For non-synonymous mutations, Hardy-Weinberg equilibrium test was performed using Fisher’s Exact test with Bonferroni corrections to determine the heterozygote deficit in each population. To determine the association between *kdr* mutations and resistance in the two populations from southern China, Fisher’s Exact test was performed and odds ratio was determined between resistant and susceptible mosquitoes for each *kdr* allele.

To determine population resistance status to pyrethroids, mortality rates of the two populations from southern China was calculated. Resistance status was classified according to WHO (2013) criteria: resistant for <90% mortality, probable resistant for 90–98% mortality, and susceptible for >98% mortality [56]. The 50% and 95% knockdown time, KDT_50_ and KDT_95_, were determined based on exponential decay model.

## Results

### Frequency of *kdr* mutations

**Fig 1:**
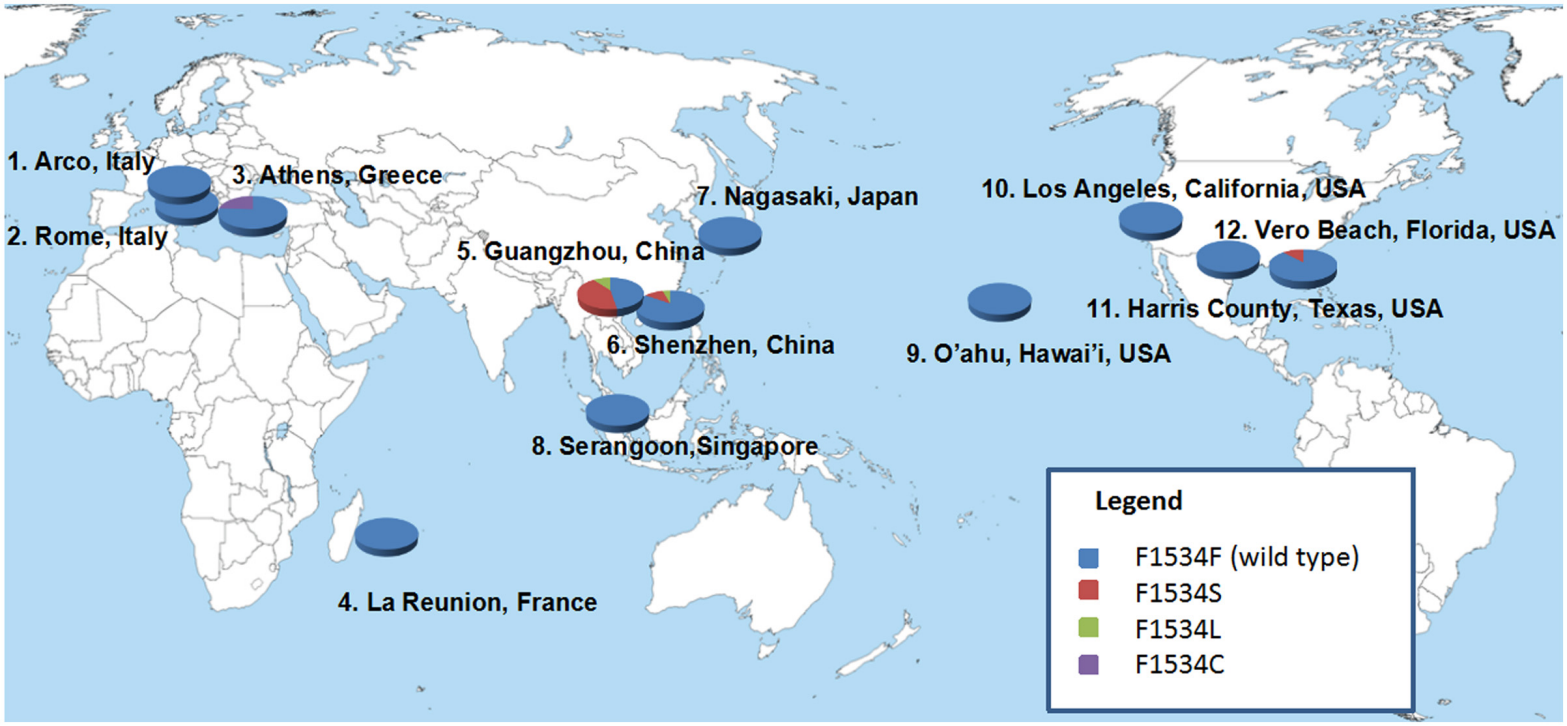
Distribution of mutation frequencies of Voltage-gated Sodium Channel gene at F1534 locus in 12 field *Aedes albopictus* populations.

Sequences of domains II (480 bp), III (exon 1; 2,347 bp), and IV (280 bp) of the VGSC gene were obtained from a total of 597 mosquitoes. All mutations in codons 989, 1011 and 1016 within domains II or IV were synonymous (codon nomenclature is based on *Musca domestica* VGSC gene according to the accepted *kdr* codon nomenclature method). In domain III non-synonymous mutations were detected at codons 1532 and 1534. At codon 1532, a change from wildtype codon ATC (isoleucine) to ACC (threonine) was detected in one population only (Rome, Italy). Thirteen out of the 40 samples were heterozygotes and one was a homozygote TT, giving an I1532T mutation frequency of 19.7%. At codon 1534, polymorphism was detected in five (Guangzhou, Shenzhen, Arco, Athens and Florida) populations out of the 12 populations examined (Table 1 and Fig 1). A total of three mutated alleles were detected. Mutations from wildtype TTC (Phe) to either TCC (Ser) or TTG (Leu) was detected in southern China (Guangzhou and Shenzhen populations), with a frequency ranging from 4.9–25.4%. Mutation from TTC (Phe) to TTG (Leu) was detected in one individual from Arco, Italy as a homozygote, giving a mutation frequency of 2.6%. Mutation from TTC (Phe) to TGC (Cys) was detected in 12 Athens individuals, of which six are heterozygous F/C and six are homozygous C/C, giving a mutation frequency of 24.2%. Mutation from TTC (Phe) to TCC (Ser) was detected in 10 individuals from Florida, of which all are heterozygotes giving a mutation frequency of 11.9%. Compared to published *kdr* mutation frequency in *Ae*. *albopictus*, the mutation frequency at the 1534 codon found in our populations, particularly those from southern China and Athens, Greece very high. Also, considerable number of homozygous mutant individuals was found (30.6% in Guangzhou, 13.7% in Shenzhen, and 11.3% in Athens).

Fisher’s Exact test found that three out of four populations (Guangzhou, Shenzhen and Greece) were not in Hardy—Weinberg equilibrium for genotypes in codon 1534. Significant departure from Hardy—Weinberg equilibrium resulted from a heterozygote deficit.

A total of 29 synonymous mutations across domain II, III and IV were recorded [S2 Table].

### Susceptibility bioassay and association with *kdr* mutations

**Fig 2:**
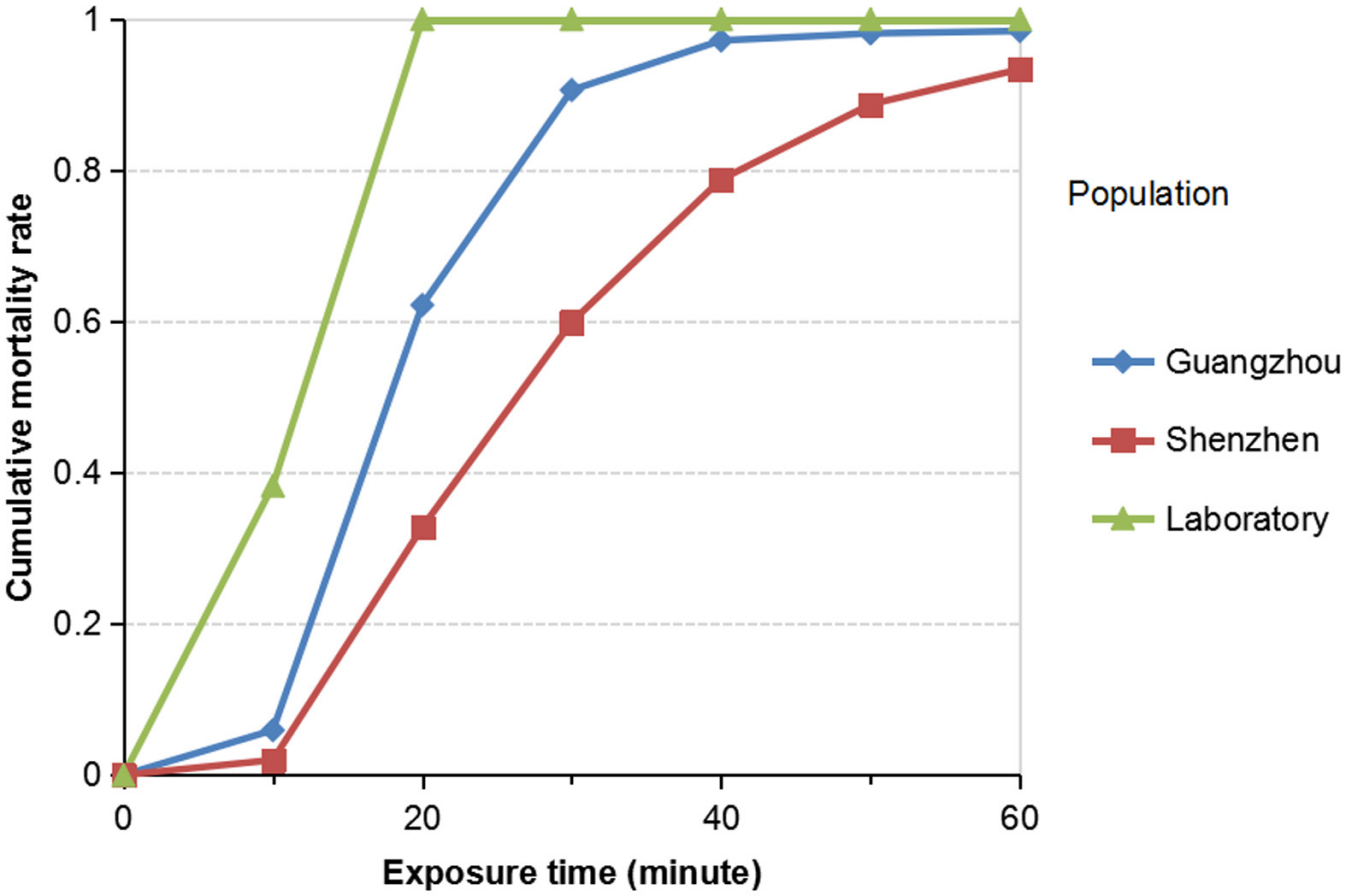
Cumulative mortality of *Aedes albopictus* against exposure time in two field population from southern China and the laboratory susceptible population.

Insecticide susceptibility bioassay found that *Ae*. *albopictus* mortality rate after the 24 hr recover period was 90.1% and 96.1% for Shenzhen and Guangzhou populations (Table 2). Based on the WHO criteria, *Ae*. *albopictus* population from Shenzhen was classified as “resistant” and the Guangzhou population as probably resistant. The 50% knockdown time (KDT_50_) was 2.3 times in Shenzhen population compare to the susceptible laboratory colony, and 1.7 for the Guangzhou population (Table 2). Similar increase in knockdown time was also found in the 95% knockdown time (KDT_50_) in field population compared to the control population. This pattern of delayed knockdown in Shenzhen and Guangzhou populations was consistent with bioassay mortality rates and population resistance classification (Fig 2).

**Table 2:** Knockdown time and mortality rate of *Aedes albopictus* populations from southern China using the standard WHO tube susceptibility bioassay against 0.05% deltamethrin.

Population	n	KDT_50_ (95% CI)	KDT_95_ (95% CI)	KRR_50_ (95% CI)	KRR_95_ (95% CI)	24 hr mortality (95% CI)
Laboratory	175	10.5 (9.5–11.5)	15.5 (13.7–20.5)	1	1	100% (n.a.) ^†^
Guangzhou	420	18.2 (16.4–19.6)	40.2 (36.4–46.5)	1.7 (1.7–1.7)	2.6 (2.3–2.7)	96.1% (94.0–98.2%)
Shenzhen	150	24.7 (19.1–29.4)	60.1 (55.5–66.1)	2.3 (2.0–2.6)	3.9 (3.2–4.1)	90.1% (83.5–96.7%)

KDT_50_: Time in minutes when 50% of the tested mosquitoes were knocked down; 95% CI refers to 95% confidence interval.

KRR_50_: Knockdown resistant ratio was calculated as KDT_50_ field population divided by KDT_50_ of laboratory strain;

KDT_95_: Time in minutes when 95% of the tested mosquitoes were knocked down;

KRR_95_: KRR_95_ was calculated as the ratio of KDT_95_ of field population to KDT_95_ of laboratory strain.

^†^ n.a., not applicable.

### Association between *kdr* mutation at 1534 codon and pyrethroid resistance

We genotyped 1534 codon of the VGSC gene for a total of 347 female mosquitoes from the two southern China populations that have been phenotyped for resistance to deltamethrin. Among them, 194 individuals were classified as “resistant” (being alive after the 24 hr recovery period in the WHO tube bioassay), and 153 were “susceptible”. Three alleles (wildtype F1534, F1534S and F1534L) and five genotypes were detected indicating two mutations at this codon (Table 3). To determine the impact of *kdr* mutation at 1534 codon on pyrethroid resistance, F1534S and F1534L alleles were analyzed separately for their associations with deltamethrin resistance. We found that F1534S mutation frequency was significantly higher in the resistant population than in the susceptible population for both Shenzhen and Guangzhou. F1534S mutation showed increased protection against deltamethrin in both populations (odds ratio for Guangzhou 3.3, *p* <0.0001; odds ratio for Shenzhen 2.7, *p* <0.0001) (Table 3). On the other hand, F1534L mutation was not significantly associated with deltamethrin resistance in both populations (*P* > 0.05).

**Table 3:** Genotyping results of voltage-gated sodium channel gene at 1534 codon and association between *kdr* mutation and phenotypic resistance in two deltamethrin resistant and susceptible *Aedes albopictus* populations from southern China.

Population	Phenotype	N	Genotypes					Odd ratio (95% CI)		Fisher Exact Probability Test: *P*	
			FF	FS	SS	FL	LL	F1534S	F1534L	F1534S	F1534L
Guangzhou	R	79	39	37	0	3	0	3.3 (2.03–5.42)	0.2 (0.08–0.47)	<0.0001	>0.05
	S	81	50	13	4	11	3				
Shenzhen	R	115	37	66	5	7	0	2.7 (1.75–4.13)	0.4 (0.18–0.73)	<0.0001	>0.05
	S	72	34	26	1	11	0				

R, Resistant; S, Susceptible; NA, not applicable.

## Discussion

The present study is by far the most comprehensive survey of *kdr* mutations in *Ae*. *albopictus* mosquitoes from broad geographical regions. Two important findings arose from this study. First, we identified two novel *kdr* mutations: I1532T and F1534S. Along domains II, III and IV of the VGSC gene, non-synonymous mutations were detected only at two codons (1532 and 1534). A novel I1532T mutation that has not been previously reported in *Ae*. *albopictus* was found uniquely present in Rome, Italy among the 12 populations examined, and it was prevalent with a frequency of 19.7%. Interestingly, this mutation was not found in the Arco population, which is 570 km away from Rome in Italy. It is worth mentioning that the mosquitoes from Arco were collected in 2011, two years prior to those collected at Rome. Hence, the difference in collection time and/or limited sample size may influence detection of this mutation at the population level. The second novel F1534S mutation was found abundant in the two populations from southern China and Florida with a frequency ranging from 9.5–22.6%. The second important finding is that distribution of *kdr* mutations in *Ae*. *albopictus* was patchy as evidenced by that fact among the 12 field populations examined, five populations exhibited polymorphism at codon 1532 or 1534, and 7 populations were monomorphic. Surprisingly modest F1534C mutation frequency was found in the Greece population. Pyrethroids were used primarily for personal protection in domestic applications in the urban environments. Ultra low volume sprays and long-term use of pyrethroids in surrounding agricultural fields may have accelerated the selection for pyrethroid resistance. However, whether the F1534C mutation can be used as a biomarker for pyrethroid resistance monitoring in *Ae*. *albopictus* populations in Greece need further evidence from pyrethroid resistance bioassay.

Using the mosquito samples from southern China, we established that *kdr* mutation conferred protection against deltamethrin in *Ae*. *albopictus* by an odds ratio of 3.3 in Guangzhou and 2.7 in Shenzhen (Table 3). This finding was consistent with studies on *Ae*. *aegypti* which reported that F1534C mutation was significantly deltamethrin resistance [25,29,32] The role of F1534C mutation in insecticide resistance was further confirmed in *Xenopus oocyte* by the demonstration of this mutation reduced the channel sensitivity to pyrethroids [32,57,58]. The present study established significant association between F1534S mutation and pyrethroid resistance in *Ae*. *albopictus* in the two south China populations. We found a modest frequency of *kdr* mutations in the two southern China populations. This modest frequency of *kdr* mutations may be related to intense pyrethroids usage in the past two decades in the area where major dengue outbreaks have occurred [26,59–64]. Pyrethroids have been the major insecticide used for city-wide aerial spray for adult mosquito control to contain dengue outbreaks [26,54,59–62,65]. Therefore, monitoring the *kdr* mutation frequency may aid the surveillance of pyrethroid resistance in *Ae*. *albopictus*.

We recognize several limitations in our study. First, only two out of 12 populations were bioassayed for pyrethroid resistance. Due to logistic constrains, it was not possible for us to collect a large number of *Ae*. *albopictus* larvae and conduct resistance bioassay. Second, survey on *kdr* mutation frequency on more countries would be ideal. Third, the association between *kdr* mutations and resistance was examined based on two populations, and the generality of the finding should be tested.

The findings from this study have important implication on *Ae*. *albopictus* control. First, the patchy distribution of *kdr* mutations in *Ae*. *albopictus* mosquitoes calls for developing global surveillance plan for pyrethroid resistance and developing countermeasures to mitigate the spread of resistance. It is entirely possible that many *Ae*. *albopictus* populations in the field are susceptible to pyrethroid, containing the spread of pyrethroid resistance would greatly preserve the effectiveness of pyrethroid insecticides. Second, significant association between *kdr* mutation and protection from deltamethrin raised the possibility that *kdr* mutation may be a viable biomarker for pyrethroid resistance surveillance in *Ae*. *albopictus*. We do not discount the potential role of metabolic detoxification enzymes and other resistance mechanisms in pyrethroid resistance, rather we emphasize that more research is needed to validate the correlation between *kdr* mutation and pyrethroid resistance at the population level.

## Supporting Information

S1 Table Detailed description of *Aedes albopictus* mosquito samples used in the study. (DOCX)

S2 Table Synonymous mutation frequency of the voltage-gated sodium channel gene domains II, III and IV in *Aedes albopictus* populations. (XLSX)
