# Supplementary material for: Multi-country Survey Revealed Prevalent and Novel F1534S Mutation in Voltage-Gated Sodium Channel (VGSC) Gene in Aedes albopictus
Source: PLoS Negl Trop Dis. 2016 May 4;10(5):e0004696. doi: 10.1371/journal.pntd.0004696 (PMC4856356; doi:10.1371/journal.pntd.0004696)
Supplement: S1 Table — (DOCX) [file pntd.0004696.s001.docx]

S 1 Table. Detailed description of *Aedes albopictus* mosquito samples used in the study.

| Site | Population | Municipality | Coordinates | Year of collection | Human density inhabitants/Km^2^ |
| --- | --- | --- | --- | --- | --- |
| 1 | Nagasaki, Japan | Nagasaki | 32.750286, 129.877667 | 2011 | 1,100 |
| 2 | Guangzhou, China | Baiyun | 23.129110, 113.264385 | 2014 | 1,100 |
| 3 | Shenzhen, China | Longgang | 22.543099, 114.057868 | 2014 | 2,200 |
| 4 | Serogoon, Singapore | Serangoon | 1.362176, 103. 870239 | 2011 | 7301 |
| 5 | La Reunion, France | Saint Pierre | -21.115141, 55.536384 | 2012 | 240 |
| 6 | Arco, Italy | Arco, Trentino | 45.917826, 10.886866 | 2011 | 78 |
| 7 | Rome, Italy | Pavia | 45.3218166, 8.846623 | 2013 | 496 |
| 8 | Athens, Greece | Thessalia | 39.6102887, 22.047637 | 2013 | 52 |
| 9 | California, USA | Los Angeles County | 33.976124, -117.905339 | 2011 | 3,198 |
| 10 | Texas, USA | Harris county | 29.775183, -95.31025 | 2011 | 759 |
| 11 | Hawai’i, USA | O’ahu | 21.447317, -158.014812 | 2011 | 632 |
| 12 | Florida, USA | Vero beach | 27.664827, -80.397274 | 2013 | 87 |
